# Clinical characteristics of the Ala21Val variant in the myelin proteolipid protein 1 (PLP1) gene associated with Pelizaeus-Merzbacher disease in a Brazilian male patient

**DOI:** 10.1038/s41439-024-00306-8

**Published:** 2025-01-06

**Authors:** Pedro Manzke, Pedro Renato P. Brandão, Talita Balieiro, Diógenes Diego de Carvalho Bispo, Maria Joana Osório, Gustavo Barcelos Barra

**Affiliations:** 1 Department of Neurology, Movement Disorders Clinic, Instituto Hospital de Base (IHB), Brasília, DF Brazil; 2 Neuroscience and Behavior Lab, University of Brasília (UnB), Brasília, DF Brazil; 3 Brasilia University Hospital, University of Brasilia (UnB), Brasília, DF Brazil; 4 Center for Translational Neuromedicine, University of Rochester Medical Center, Rochester, NY USA; 5 Center for Translational Neuromedicine, University of Copenhagen, Copenhagen, Denmark; 6 Sabin Diagnóstico e Saúde, Brasília, DF Brazil

**Keywords:** Movement disorders, Neuromuscular disease

## Abstract

Here, we report the case of a 29-year-old male with classic Pelizaeus-Merzbacher disease (PMD) harboring the *PLP1* variant NM_000533.5:c.62 C > T, leading to an NP_000524.3:p.(Ala21Val) alteration in the first transmembrane domain of the protein. He presented with developmental delays, nystagmus, spastic paraparesis, optic atrophy, dysphagia, appendicular ataxia, and progressive head tremor. Brain MRI revealed hypomyelination, diffuse white matter hyperintensity, and atrophy of the corpus callosum and cerebellum, expanding the known clinical spectrum of PMD.

Pelizaeus-Merzbacher disease (PMD) is an X-linked leukodystrophy that is characterized by developmental delay, nystagmus, hypotonia, spasticity, and varying levels of intellectual disability. This condition is caused by variants in the PLP1 gene, including duplications, deletions, and point mutations^1^. Despite its monogenic origin, PMD shows substantial clinical variability. Patients are categorized into five phenotypic grades on the basis of their highest motor abilities, ranging from Form 0 to Form 4^2^. The most severe phenotypes, Forms 0 and 1 (connatal PMD), are associated with early onset and severe motor and cognitive deficits, often accompanied by dystonia, seizures, and other neurological symptoms. Patients with milder phenotypes, such as Forms 2 and 3 (classic PMD), demonstrate greater motor abilities but still present with hypotonia^3^. While nystagmus may decrease as the disease progresses, other motor and cognitive impairments tend to worsen^4^. The mildest phenotype, spastic paraplegia 2 (SPG2) or Form 4, is characterized primarily by progressive spasticity in the lower limbs^5^. PLP1 gene duplications are typically associated with Forms 1 and 2, whereas point mutations in PLP1 can lead to a spectrum of phenotypes, from the most severe (Form 0) to the mildest (Form 4)^2,6^. Individuals with deletions or nonsense/frameshift mutations that result in null alleles usually present with milder phenotypes, primarily Forms 3 and 4^7^.

The proband, a 29-year-old Brazilian man, was evaluated at the Movement Disorders Clinic in the Hospital de Base do Distrito Federal. His symptoms began in early childhood with cervical hypotonia, followed by horizontal nystagmus and convergent strabismus. By nine months, he could hold his head up, and by one year, he could sit without support, but he never walked unaided. The patient’s speech development was incomplete, and dysphagia emerged at age five. Owing to intellectual disability, he attended a special school and exhibited behavioral changes, such as irritability, aggression, and mood swings. Three years before evaluation, a worsening horizontal head tremor appeared, further complicating his condition. These clinical manifestations were indicative of Form 3 of the severity classification system proposed by Cailloux et al.^2^.

On physical examination, a small café-au-lait spot was noted on his left thigh. Cranial nerve assessment revealed convergent strabismus due to lateral rectus muscle paresis, inconsistent vertical upward gaze, and pendular nystagmus. The patient exhibited appendicular ataxia, dysdiadochokinesia affecting all limbs, and pronounced spastic paraparesis. Bilateral cutaneous–plantar reflex extension and Achilles clonus indicated pyramidal tract involvement, whereas sensory examination revealed no abnormalities. Vision was subnormal in both eyes due to optic atrophy, although hearing was normal. Swallowing video endoscopy revealed involuntary vocal cord movements, and abdominal ultrasound revealed no liver or spleen abnormalities.

Magnetic resonance imaging (MRI) using a 1.5-Tesla scanner with FLAIR, T1, and T2 sequences and proton MR spectroscopy revealed brain volume loss, with atrophy of the corpus callosum and cerebellum. T2 and FLAIR images revealed diffuse hyperintensity in the periventricular, deep, and subcortical white matter; internal capsules; and corpus callosum, with signal preservation in most of the brainstem. Proton MR spectroscopy of the right parietal white matter revealed a decreased NAA/Cr ratio and increased mI/Cr ratio, indicating hypodysmyelination (Fig. 1).

**Fig. 1:**
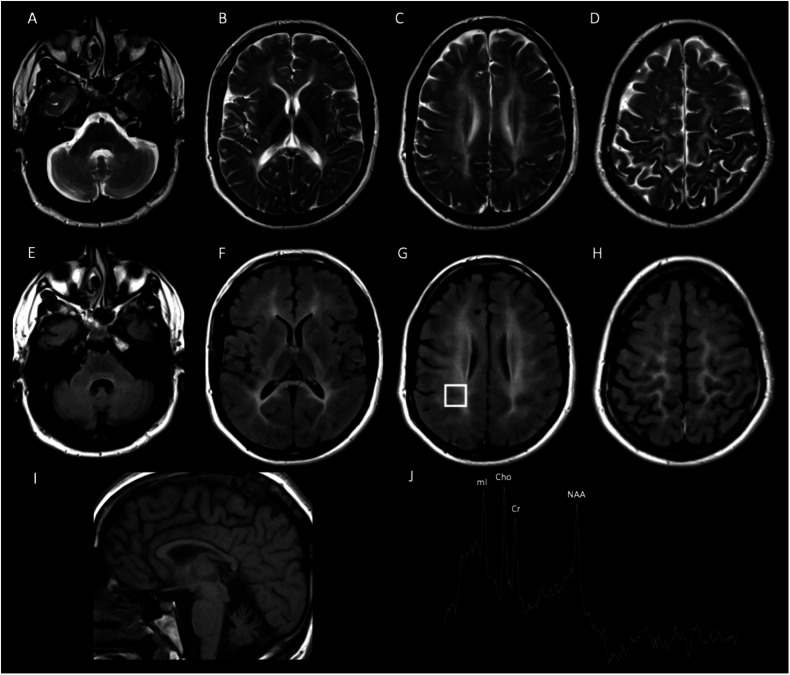
Brain MR image of a 29-year-old male proband. Axial T2-weighted (**A**–**D**) and FLAIR (**E**–**H**) images showing hyperintensity in the white matter, internal capsules, and brainstem. Sagittal T1-weighted image (**I**) revealing cerebral and cerebellar atrophy, including the corpus callosum. Proton MR spectroscopy (**J**) revealed decreased NAA/Cr ratios and increased mI/Cr ratios, indicating hypodysmyelination.

DNA was extracted from the patient’s peripheral blood and sequenced targeting the coding exons of approximately 4000 genes associated with inherited diseases. These genes were enriched using the KAPA Hyperplus Library Preparation Kit, the SeqCap EZ Share Choice—Inherited Disease Panel, and the SeqCap EZ Hyper Prep Kit (all from Roche), followed by paired-end sequencing with 2 × 75 bases on an Illumina NextSeq 500 platform. FastQ files were processed with DRAGEN Germline 3.5.7 for alignment and variant calling, using GRCh37/hg19 as the reference genome. Variant annotation was conducted with Varstation. The mean coverage was 104X, and the sequencing uniformity was 99.24%. Genes related to leukodystrophy and leukoencephalopathy were analyzed.

A missense variant in the *PLP1* gene, NM_000533.5:c.62 C > T/NP_000524.3:p.(Ala21Val), located at chrX-103040568-C-T, was identified in a hemizygous state (Fig. 2A). Sanger sequencing was utilized for variant confirmation and family segregation analysis. Cosegregation analysis revealed that the proband inherited the variant from his mother, who is heterozygous for the variant. This variant was not detected in other maternal relatives (aunt, uncle, or two cousins). The mother presents with tremors, resembling essential tremors, with noticeable head and upper limb tremors.

**Fig. 2:**
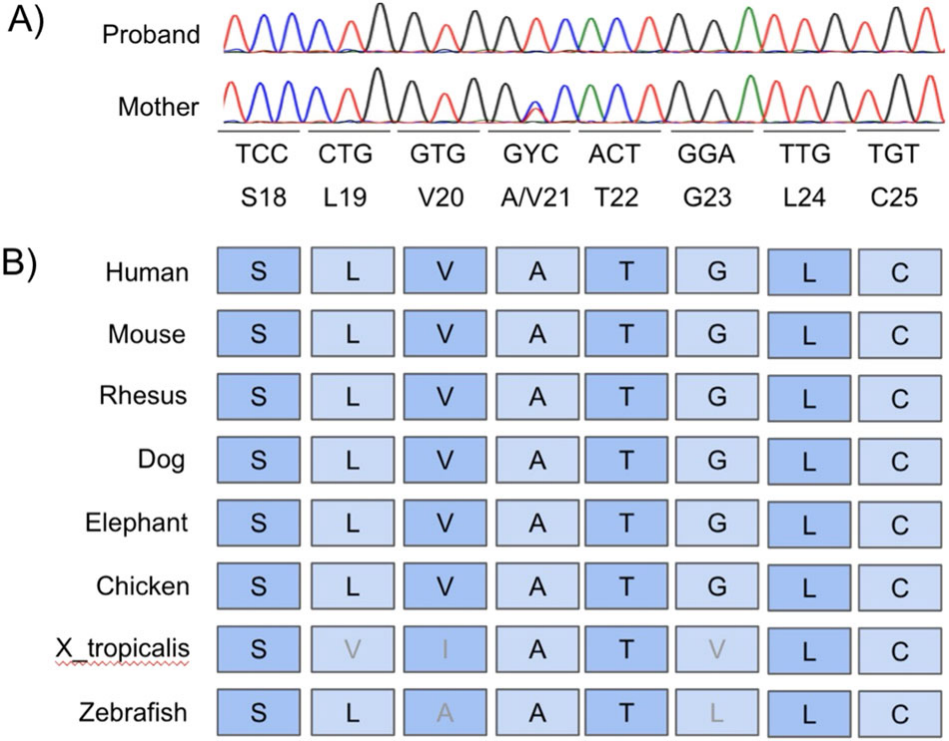
PLP1 Sequencing. **A** Sanger sequencing showing the PLP1 variant (NM_000533.5:c.62 C > T (p.Ala21Val)) in the proband, which was inherited from his mother but was absent in other maternal relatives. ‘Y’ denotes C or T nucleotides. **B** Alignment of the PLP1 sequence around the mutation, highlighting the conserved alanine 21 in the transmembrane domain across species.

This *PLP1* variant (NM_000533.5:c.62 C > T/NP_000524.3:p.(Ala21Val)) has not been reported in major population databases, including gnomAD, ExAC, and ABraOM, which satisfies ACMG rule PM2 (moderate). Eleven computational tools, including Revel (deleterious, 0.96), unanimously predicted a deleterious effect, which satisfies rule PP3 (strong). Pathogenic *PLP1* missense variants significantly outnumbered benign variants in ClinVar, which satisfies rule PP2 (supporting). The variant is located within the first transmembrane domain (residues 12 to 40, UniProt P60201); 31 variants have been identified in this domain, 23 of which are pathogenic or likely pathogenic^8^, which satisfies rule PM1 (moderate). Cosegregation analysis revealed no hemizygous or homozygous relatives, but this result had minimal impact on the interpretation because of the X-linked inheritance pattern. This variant was previously described in a case series and classified as likely pathogenic, but the affected patient was lost to follow-up, and no clinical characteristics were documented^3^. The high conservation of the mutated residue and the patient’s phenotype satisfy rule PP4 (supporting). Collectively, these findings classify the variant as likely pathogenic^9,10^.

In conclusion, the patient’s clinical and MRI findings confirmed a diagnosis of classic PMD, which was caused by the PLP1 variant NM_000533.5:c.62 C > T/NP_000524.3:p.(Ala21Val). The early onset of hypotonia and nystagmus, followed by ataxia and optic atrophy, is consistent with previously reported cases^11,12^. Although significant lower limb spasticity suggests a possible SPG2 phenotype, this diagnosis is unlikely. This case adds to the evidence supporting the pathogenicity of missense variants in the PLP1 transmembrane domain.

## HGV database

The relevant data from this Data Report are hosted at the Human Genome Variation Database at 10.6084/m9.figshare.hgv.3453.
